# GLP-1 receptor agonists in Parkinson’s disease: an updated comprehensive systematic review with meta-analysis

**DOI:** 10.1186/s13098-025-01888-1

**Published:** 2025-08-23

**Authors:** Mohamed Mohsen Helal, Hala AbouShawareb, Omarfayez Hussein Abbas, Roaa Haddad, Youmna Zain, Ahmed S. A. Osman, Amr K. Hassan

**Affiliations:** 1 Faculty of Medicine, Zagazig University, Zagazig, Egypt; 2 Faculty of Medicine, Mansoura University, Mansoura, Egypt; 3 Faculty of Medicine, Al-Azhar University, Cairo, Egypt; 4 Faculty of Medicine, October 6 University, Giza, Egypt; 5 Faculty of Medicine, Tanta University, Tanta, Egypt; 6 Faculty of Medicine, Minia University, Minia, Egypt; 7 Department of Ophthalmology, School of Medicine, University of California, Irvine, CA USA; 8 Medical Research Group of Egypt, Negida Academy, Arlington, MA USA

**Keywords:** Glucagon-like peptide-1 receptor agonists, Parkinson disease, Motor disorders, Humans, Randomized controlled trials

## Abstract

Previous studies have demonstrated an increased risk of developing Parkinson’s disease (PD) in patients with type 2 diabetes mellitus (T2DM), as well as more severe and rapid motor and non-motor deterioration in diabetic PD patients compared to their non-diabetic counterparts. Additional research has suggested that diabetic subjects treated with glucagon-like peptide-1 (GLP-1) receptor agonists exhibit a reduced incidence of PD compared to those receiving other anti-diabetic medications. GLP-1 receptor agonists are FDA-approved therapies for T2DM, and recent studies have explored their potential as repurposed treatments for neurodegenerative diseases, including PD, AD, and ALS, as well as cerebrovascular disorders. This systematic review aims to assess the available literature on the efficacy and safety profiles of GLP-1 receptor agonists in PD management. A comprehensive search of PubMed, Scopus, CENTRAL, Web of Science, Embase, and ClinicalTrials.gov was conducted to identify relevant studies. The primary outcomes of this review include motor impairment in PD, as assessed by MDS-UPDRS Part III, as well as motor complications (Part IV) and motor experiences of daily living (Part II), and the incidence of gastrointestinal and systemic side effects. Meta-analysis showed that GLP-1 receptor agonists significantly improved motor function, as reflected by MDS-UPDRS Part III scores in the ON state (mean difference = − 2.88; p = 0.01; I^2^ = 30%), although they were associated with a higher incidence of adverse events across all safety outcomes. Findings and conclusions of this review will contribute to a clearer understanding of the therapeutic potential of GLP-1 receptor agonists in PD, guiding future clinical research and treatment strategies.

## Background

Globally, neurological disorders have emerged as a leading cause of disability, particularly with the aging population, contributing to an increase in neurodegenerative diseases. Among these, Parkinson's disease (PD) stands out as the second most prevalent neurodegenerative disorder [1]. A meta-analysis conducted in 2016 revealed a staggering 74% increase in the prevalence of PD from 1990 to 2016 [2]. This was not attributed only to the increased number of the aging population, since age-standardized prevalence has significantly escalated as well.

PD is primarily characterized as a movement disorder, stemming from dopamine depletion in the brain due to the degeneration of dopamine-producing neurons in the substantia nigra (SN), which are crucial for movement coordination. This manifests by resting tremors, stiffness, and imbalance, among others. Various hypotheses have emerged regarding the underlying mechanisms of neuroinflammation and degeneration, including the abnormal clearance of α-synuclein protein in affected neurons, mitochondrial dysfunction, and oxidative stress [3].

Impaired glucose metabolism in PD patients exists in the early stages of the disease [4]. This impairment may be attributed to various mechanisms, such as insulin resistance, oxidative stress, and blood–brain barrier dysfunction [5]. Impaired insulin signaling can lead to mitochondrial dysfunction that is characterized by compromised energy metabolism, which is detrimental to neuronal health. It’s also believed to increase the aggregation of α-synuclein, which is highly toxic to the neurons [6]. Insulin resistance also contributes to increased production of reactive oxygen species, further leading to oxidative stress that damages dopaminergic neurons in the SN.

Currently, there are no disease-modifying therapies for PD. However, new studies continue to offer insights into optimal symptomatic management [7]. A growing body of evidence is studying the relationship between neurodegenerative diseases such as PD and type 2 diabetes mellitus (T2DM). Cohort studies have shown an increased risk of developing PD in individuals with diabetes [8]. These findings align with the aforementioned mechanisms of insulin resistance in promoting the pathologies of PD. Furthermore, studies indicate that diabetics treated with glucagon‐like peptide‐1 (GLP‐1) receptor agonists have a *reduced risk* of PD compared to those on other anti-diabetic medications, suggesting possible neuroprotective effects [9, 10].

GLP-1 receptor agonists are FDA-approved treatments for T2DM and act by triggering insulin release by stimulating the GLP-1 receptor in the pancreas. Their recognized *neuroprotection* in PD models is thought to arise from their promotion of survival of dopaminergic neurons in the SN. This effect is presented through several mechanisms, such as reducing oxidative stress, apoptosis, and inflammation [11, 12]. Additionally, these agents have been found to resensitize insulin signaling in the brain, potentially addressing metabolic impairments associated with PD [13].

Clinical trials investigating GLP-1 receptor agonists in PD patients have yielded mixed results regarding their impact on both motor and non-motor symptoms of the disease as well as related adverse events. Given their promising efficacy in addressing PD pathologies, it is vital to evaluate both the magnitude and direction of their effects on clinically significant PD symptoms. Consequently, this systematic review and meta-analysis aims to assess the safety and efficacy of GLP-1 receptor agonists for individuals suffering from PD.

## Methods

We followed the Preferred Reporting Items of Systematic Reviews and Meta-Analysis (*PRISMA*) statement guidelines to report this review [14]. All steps were done in strict accordance with the *Cochrane Handbook for Systematic Reviews of Interventions version 6.4* [15]. This review was registered a priori on the International Prospective Register of Systematic Reviews (PROSPERO) under the *ID CRD42024594452*.

### Criteria for considering studies for this review

#### Types of studies

We aimed to gather present evidence that investigates the role of GLP-1 receptor agonists in PD patients. We considered studies that are ongoing or completed, published or unpublished, and trials that are randomized or nonrandomized for inclusion in this review. The inclusion of *ongoing studies* (summarizing their characteristics and including them in the synthesis of evidence) and unpublished trials is recommended by methodological guidelines and stated by *Cochrane Handbook* [15]. For unpublished trials, we searched trial registries (such as clinicaltrials.gov) and preprint websites and obtained results of an included trial in this review through a combination of both [16].

If a trial is reported in more than one report, we included all reports and extracted data from them all while paying close attention so as not to report duplicated data. These reports could be preprints, journal articles of the main results, and post hoc analyses [17]. We did not prioritize a type of published report to be extracted if there was a conflict between reports and resolved these conflicts by discussion to reach the most appropriate data.

There was no restriction for publication language, year of dissemination, or setting. As stated by methodological guidelines, reported outcomes in the trials were not used as eligibility criteria [18]. Cross-over trials were intended to be excluded since the applicability of their results in PD is doubtful; however, our search did not recall any such trials related to the topic [15].

#### Participants

We included subjects diagnosed with PD with any validated tool, with no restriction of age, disease stage, or number of participating patients in a trial.

#### Types of interventions

GLP-1 receptor agonists are the intervention of interest in this review, with no restrictions on dose, route of administration, duration of treatment, or specific drug in this family. Comparators were control groups (groups receiving no treatment or usual care) or placebo groups. Comparators are referred to as “placebo” in the plots.

### Types of outcome measures

#### Primary outcomes

Primary outcomes included PD motor impairment as measured by the Movement Disorder Society-Unified Parkinson's Disease Rating Scale (MDS-UPDRS) subscale Part III in the on-medication state in endpoints and midpoints of included studies and in the off-medication state in endpoints and midpoints of included studies [19]. Other primary outcomes included PD motor complications as measured by MDS-UPDRS Part IV in endpoints of included studies, motor experiences of daily living in PD patients as measured by MDS-UPDRS Part II in endpoints of included studies, gastrointestinal adverse events of GLP-1 receptor agonists, including dyspepsia, vomiting, diarrhea, and constipation, as well as general adverse events such as headache and fatigue.

#### Secondary outcomes

Secondary outcomes included non-motor symptoms of PD as measured by the Non-Motor Symptoms Scale (NMSS), quality of life of PD patients as measured by the Parkinson’s Disease Questionnaire (PDQ)‐39, and changes in levodopa equivalent doses (LED) from baseline to endpoints.

Choosing *motor outcomes* to be included as critical outcomes in the comparison of GLP-1 receptor agonists vs. control or placebo groups was based on their significance in the lives of individuals with PD in accordance with Hammarlund et al., who stated these are the most important outcomes [20]. In addition, adverse events were of such interest in this review to complete the efficacy and safety profiles of GLP-1 receptor agonists in PD treatment. Furthermore, irrespective of the importance of these outcomes, the outcomes of quality of life of subjects with PD, non-motor symptoms, and required daily doses of medications were considered to be of clinical importance for them to maintain a more convenient life with fewer drug side effects.

### Search methods for identification of studies

#### Electronic searches

A comprehensive search of electronic databases, including PubMed, Scopus, Cochrane Central Register of Controlled Trials (CENTRAL) in the Cochrane Library, Web of Science, and Embase, has been performed on 9/9/2024 for articles relating to PD and GLP-1 receptor agonists with keywords that include the following: ((Parkinson) OR (Parkinson*) OR (Parkinson's)) AND ((Glucagon) OR (GLP-1) OR (Glucagon-like peptide-1) OR (Dulaglutide) OR (Exenatide) OR (Liraglutide) OR (Lixisenatide) OR (Semaglutide) OR (Tirzepatide)).

#### Searching other resources

We searched the internet for more relevant studies to include. This included searching the trials registry at clinicaltrials.gov. Moreover, searching for comments or errata for the included studies was done [21]. We hand-searched the included studies for other eligible ones. The hand search includes backward and afterward searches [22]. In the backward search, we searched the listed references of included studies, while in the afterward search, we used websites such as Google Scholar and https://www.uww.edu/library (the University of Wisconsin–Whitewater’s library website) to identify where each of the included studies was cited.

### Data collection and analysis

#### Selection of studies

Studies retrieved from different databases were merged together, then duplicates were deleted using the reference management software EndNote Web [23]. Following this, results were uploaded to Rayyan software [24] to conduct the screening process of the literature search results. Studies were screened in two rounds. The first was a blinded title and abstract screening. In the second phase, a full-text article screening of the potentially eligible abstracts was conducted. Each study was judged by two independent authors, and any disagreements were resolved by consulting a third reviewer. We excluded studies that did not match our model of population and intervention under investigation.

#### Data extraction and management

We used a customized Google spreadsheet for data extraction that was accessible to all authors. All reviewers took part in the data extraction. Data from each study was extracted independently by two authors. Any disagreements were resolved through discussion between the two reviewers, and sometimes a third reviewer participated. Extracted data were mainly divided into five domains: study characteristics, characteristics of the included studies’ population, risk of bias domains, and results of studies, including results of the efficacy profile and adverse events. Characteristics of the included studies included study ID (last name of the first author and the publication year), study design, country, sample size, intervention, and comparator. Characteristics of the population included the assigned intervention, age (years), sex (male, %), weight (kg), body mass index (BMI, kg/m2), time since diagnosis (years), baseline MDS-UPDRS Part III on-state, MDS-UPDRS Part III off-state, and MDS-UPDRS Part IV, and MDS-UPDRS Part II.

Some data in the trials’ reports were represented in graphs, and two reviewers independently used PlotDigitizer [25] for its extraction. As we were looking forward to comparing the efficacy of GLP-1 receptor agonists in midpoints and endpoints, data from these time points were extracted separately. The midpoint of interest was 6 months after the application of the intervention, and the endpoint of interest was the actual endpoint in the included studies. Table 1 shows the timepoints when data were obtained from patients in the included studies. Continuous data that was not reported as mean and standard deviation (SD) was converted to this format using the Meta-Analysis Accelerator website [26].

**Table 1:** Timepoints of outcome assessments in the included trials [27–31]

Study name	Intervention	Timepoint 1	Timepoint 2	Timepoint 3	Timepoint 4	Timepoint 5	Timepoint 6
Athauda 2017	Exenatide	12 weeks	24 weeks*	36 weeks	48 weeks	60 weeks^#^	–
Aviles-Olmos 2013	Exenatide	26 weeks*	48 weeks	52 weeks^#^	56 weeks	–	–
Malatt 2022	Liraglutide	28 weeks*	54 weeks^#^	–	–	–	–
McGarry 2024	NLY01 2.5, 5 mg	4 weeks	8 weeks	12 weeks	24 weeks*	36 weeks	44 weeks^#^
Meissner 2024	Lixisenatide	26 weeks*	52 weeks^#^	–	–	–	–

^*^: Timepoints used as midpoints and pooled together

^#^: Timepoints used as endpoints and pooled together

#### Assessment of risk of bias in included studies

The quality of the studies included in this review was assessed by two independent reviewers using the Cochrane risk of bias tool 2 (RoB 2) [32]. The Cochrane RoB 2 has seven study domains, including random sequence generation, allocation concealment, blinding of the investigators and participants, blinding of the outcome assessors, incomplete outcome data, selective outcome reporting, and other sources of bias. For each domain, studies were categorized as having a “low risk,” “high risk,” or “some concerns” of bias.

#### Measures of treatment effect

To calculate the pooled effect estimates of efficacy outcomes of GLP-1 receptor agonists vs. controls, continuous data was analyzed based on mean and SD with the total number of participants in each study group (N) to calculate the mean difference (MD) with 95% confidence interval (CI). Standardized mean difference (SMD) was not used as the studies used the same scale in each outcome domain. In addition, risk ratios (RR) were used to express the pooled effects of GLP-1 receptor agonists vs. placebo regarding adverse events of their application. Dichotomous data was extracted to calculate RRs.

#### Unit of analysis issues

Our unit of analysis was the PD patient. One included study had three arms: two arms for the drug with different doses and one arm for placebo. In each outcome, we combined the results of the two drug arms [33]. We did not choose to extract data of only one arm in order not to arbitrarily omit a relevant group to our research question [33]. Also, we did not include both arms in one meta-analysis separately in order not to double count the placebo group, which would raise unit-of-analysis issues in our synthesis [33]. Moreover, combining both arms of the drug is justified best in this case by our research question, which investigates the overall effect of the GLP-1 receptor agonists in PD treatment, not to compare the effect of different doses of one drug in this drug family.

#### Dealing with missing data

For potentially measured but unreported data that are of our interest in included trials, we emailed the authors of these trials to ask for it. Additionally, one included study in this review (*Malatt 2022*) does not have a full-text journal article. However, the registry of the trial on ClinicalTrials.gov had the baseline characteristics of participants and detailed results, and it has a preprint with full data [34]. Moreover, this trial is reported in an abstract in *Neurology journal* [16]. We collated data from all these sources [17].

#### Synthesis methods

We conducted a meta-analysis to demonstrate the pooled effect estimates using Review Manager (RevMan) version 5.4.1 [35]. We intended to use a random-effect meta-analysis model in efficacy outcomes comparisons since the interventions in studies are not the same drug, which explains heterogeneity if it is noted. We, however, used a fixed-effect model in the adverse events meta-analyses since adverse events of GLP-1 receptor agonists are profoundly similar. Pooled effect measures for continuous outcomes were intended to be reported using the inverse variance method, and for dichotomous outcomes, the Mantel–Haenszel methods.

#### Subgroup analysis

We aimed to conduct a subgroup analysis based on the time since diagnosis with PD (early and late subgroups) when sufficient data exists.

#### Sensitivity analysis

In the MDS-UPDRS Part III outcome domain, sensitivity analysis was intended to assess the robustness of the evidence. This was done multiple times, excluding a different study in each instance.

## Results

### Description of studies

#### Results of the search

**Fig. 1:**
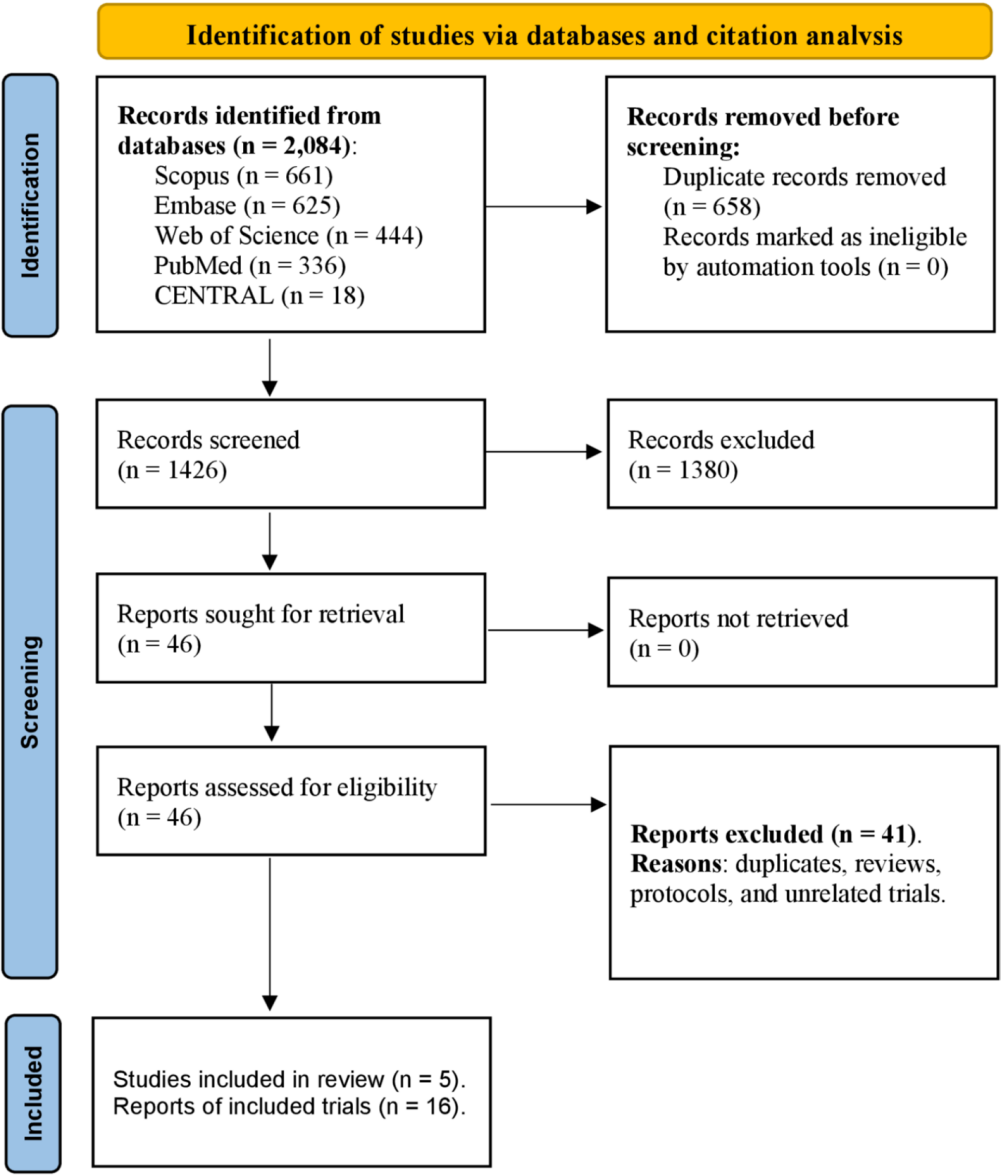
PRISMA flow diagram of the review

The search of PubMed, Scopus, CENTRAL, Web of Science, and Embase yielded 2084 entries. After removing duplicates and screening the titles and abstracts of 1426 records, 46 studies were identified as potentially relevant and underwent full-text review. Of these, only five trials met the inclusion criteria and were included in the final analysis [16, 30, 31, 36, 37]. We also identified five ongoing studies on the review topic. Figure 1 presents the PRISMA flowchart, detailing the study selection process and reasons for the exclusion of studies during the full-text screening phase.

#### Included studies

Five studies reported in 16 reports were included in the synthesis. Athauda 2017 was reported in five reports, including 3 reports of post-hoc analyses [27, 36, 38–40]. In addition, Aviles-Olmos 2013 has 6 records [28, 37, 41–44]. Malatt 2022 is described in three reports [16, 29, 34]. The included clinical trials in the quantitative synthesis were published between 2013 and 2024, involving a total of 580 patients with PD. These studies were conducted in various countries, including the United States, the United Kingdom, and France. All studies were double-blind randomized controlled trials (RCTs), except for the study by Aviles-Olmos [28], which was a single-blind RCT. In these trials, participants were randomized to receive a GLP-1 receptor agonist intervention at various doses and placebo plus usual care or standard care only as control groups. The GLP-1 receptor agonists used in these trials included exenatide, liraglutide, NLY01 (a pegylated analogue of exenatide), and lixisenatide.

A summary of the included study characteristics is presented in Table 2, while the baseline characteristics of the participants are shown in Table 3.

**Table 2:** Summary of the included studies

Study ID	Study design	Registration (NCT)	Follow‐up duration (w)	Country	N randomized	Type of Analysis	Diagnostic criteria	Intervention (s)	Comparator	Outcomes assessed	Findings
Athauda 2017 [36]	Double-blind RCT	NCT01971242	60	UK	62	ITT	Queen Square Brain Bank criteria	S.c. exenatide 2 mg once weekly + usual care	Placebo	MDS-UPDRS part III, NMSS, PDQ-39, LED, MADRS, MDRS, and Unified Dyskinesia Rating Scale	Exenatide had positive, sustained effects on motor and non-motor scores in PD patients
Aviles-Olmos 2013 [37]	Single-blind RCT	NCT01174810	52	UK	44	PP	Queen Square Brain Bank criteria	S.c. exenatide 5 μg or 10 μg BID + usual care	Usual care	MDS-UPDRS part III, part IV, part II, part I, PDQ-39, LED, MADRS, MDRS, Dyskinesia Rating Scale, and safety	Exenatide had clinically relevant improvements in PD across motor and cognitive measures
Malatt 2022 [16]	Double-blind RCT	NCT02953665	54	US	63	PP	UK Brain Bank Criteria	S.c. Liraglutide 0.6–1.8 mg daily + usual care	Placebo + usual care	MDS-UPDRS part III, part IV, part II, part I, NMSS, PDQ-39, LED, MDRS, and safety	Liraglutide showed no significant effect on motor symptoms but improved non-motor symptoms, unlike placebo, which worsened them
McGarry 2024 [30]	Double-blind RCT	NCT04154072	44	US	255	ITT	UK Brain Bank Criteria	S.c. NLY01 2.5 mg, NLY01 5 mg once weekly	Placebo	MDS-UPDRS part III, part II, and part I, NMSS, PDQ-39, SE-ADL, MoCA, SCOPA-Cog, and safety	NLY01 at 2.5 and 5 mg was not associated with any improvement in PD motor or non-motor features compared with placebo
Meissner 2024 [31]	Double-blind RCT	NCT03439943	52	France	156	ITT	UK Brain Bank Criteria	S.c. lixisenatide 10 μg or 20 μg daily + usual care	Placebo + usual care	MDS-UPDRS part III, part IV, part II, part I, LED, and safety	Lixisenatide therapy resulted in less progression of motor disability than placebo but was associated with gastrointestinal side effects

*ITT* Intention to treat, *PP* Per protocol, *RCT* randomized clinical trial, *S.c.* subcutaneous, *N* Sample size, *MDR-UPDRS* Movement Disorder Society-Sponsored Revision of the Unified Parkinson’s Disease, *MMSE* Mini-Mental State Examination, *MoCA* Montreal Cognitive Assessment, *PDQ-39* Parkinson’s Disease Questionnaire

**Table 3:** Baseline characteristics of the included studies’ populations

Study ID	Interventions	Age in years (SD)	Male N (%)	Weight in kg (SD)	BMI in kg/m^2^ (SD)	Time since diagnosis (year)	Age at PD onset in years (SD)	LED	MDS-UPDRS part III on state	MDS-UPDRS part III off state	MDS-UPDRS part II	MDS-UPDRS part IV
Athauda 2017 [36]	Exenatide	61.6 (8.2)	22 (71%)	81.8 (16.6)	–	6·4 (3.3)	55.9 (7.9)	773.9 (260.9)	19.4 (8.4)	32.8 (9.7)	12.5 (6.7)	4.7 (3.1)
	Placebo	57.8 (8.0)	22 (76%)	80.8 (12.9)	–	6·4 (3.3)	52.2 (7.7)	825.7 (215)	14.4 (8.2)	27.1 (10.3)	10.7 (5.3)	5.3 (3.0)
Aviles-Olmos 2013 [37]	Exenatide	61.4 (6.0)	15 (75%)	–	–	9.6 (3.4)	51.6 (7.8)	973 (454)	23.5 (6.3)	31.0 (11.2)	10.2 (5.2)	6.3 (2.4)
	Usual care	59.4 (8.4)	20 (83%)	–	–	11.0 (5.9)	48.4 (7.4)	977 (493)	25.3 (10.7)	34.0 (16.1)	12.9 (6.2)	6.3 (3.4)
Malatt 2022 [16]	Liraglutide	63.5 (9.8)	25 (67.6%)	–	28 (5.2)	4.7 (3.1)	58.9 (10.5)	564 (327)	14.8 (7.1)	26.1 (9.6)	8.8 (5.4)	3.8 (3.3)
	Placebo	64.2 (6.4)	13 (72.2%)	–	27.9 (4.6)	4.8 (3.3)	59.3 (7.5)	640 (360)	16.3 (9.2)	28.8 (10.7)	7.6 (5.0)	3.6 (3.2)
McGarry 2024 [30]	NLY01 2.5 mg	62.1 (9.0)	60 (71%)	80.8 (16.6)	26.42 (4.14)	1.02 (1.04)	–	–	–	22.7 (8.1)	4.8 (3.6)	–
	NLY01 5 mg	60.6 (10.0)	54 (64%)	79.1 (17.4)	25.81 (4.58)	0.96 (0.94)	–	–	–	22.0 (8.2)	5.0· (4.1)	–
	Placebo	61.8 (8.1)	52 (62%)	77.8 (16.2)	26.03 (4.66)	0.9 (0.996)	–	–	–	22·3 (9.1)	4.9 (3.6)	–
Meissner 2024 [31]	Lixisenatide	59.5 (8.1)	44 (56)	75 (15.8)	25.6 (3.9)	1.4 (0.8)	–	317 (179)	–	14.8 (7.3)	5.0 (3.5)	0.3 (1.3)
	**Placebo**	59.9 (8.4)	48 (62)	76.7 (15.7)	25.8 (4.2)	1.4 (0.7)	–	355 (215)	–	15.5 (7.8)	5.4 (4.3)	0.2 (0.8)

All qualitative variables are represented as number (percentage), while quantitative variables are shown in Mean (Standard deviation)

*LED* levodopa equivalent dose, *MDR-UPDRS* Movement Disorder Society-Sponsored Revision of the Unified Parkinson’s Disease, *SD* Standard deviation

#### Ongoing studies

Our search has detected five ongoing trials that aim to assess the effects of GLP-1 receptor agonists in individuals with PD. Exenatide has predominated the intervention groups across trials, which is justified by the favorable results from the completed trials (see Table 2). A summary of their status, interventions, follow-up durations, and main outcomes is shown in Table 4. These ongoing trials vary in their methodologies, outcomes of interest, and even in how outcomes are assessed. Once completed and published, they will yield reasonable evidence about the true evaluation of GLP-1 receptor agonists in PD.

**Table 4:** Summary of ongoing studies

PI	NCT	Status^a^	N^b^	Medication	Follow up	Main outcomes	Results
Vaillancourt [45]	NCT03456687	Completed	5	Exenatide	1 year	Change in free-water accumulation in the substantia nigra Change in BOL signal in the posterior putamen Change in BOL signal in M1 Change in BOL signal in the SMA	NA
Foltynie [46]	NCT04232969	Active, not recruiting	194	Exenatide	2 years	MDS-UPDRS Part III (ON state) MDS-UPDRS Part IV and II (OFF state) PHQ-9 PDQ-39 NMSS	NA
Min Ho Ihm [47]	NCT04269642	Unknown	99	Exenatide SR	60 weeks	MDS-UPDRS Part III Specific to non-specific binding ratio (confirmed by PET scan) MDS-UPDRS Part I, II, and IV PDQ-39 MoCA NMSS	NA
NA [48]	NCT03659682	Not yet recruiting	120	Semaglutide	48 months	MDS-UPDRS Part III (OFF state) Levodopa equivalents Nigrostriatal degeneration (assessed by changes in DAT-scan uptake) Cognitive function	NA
Svenningsson [49]	NCT04305002	Unknown	60	Exenatide	21 months	MDS-UPDRS Part III (ON and OFF states) MDS-UPDRS II and IV Accelerometer (intensity of physical activity) Accelerometer (steps per day)	NA

*BOL* Blood Oxygen Level-dependent, *MDR-UPDRS* Movement Disorder Society-Sponsored Revision of the Unified Parkinson’s Disease, *MMSE* Mini-Mental State Examination, *MoCA* Montreal Cognitive Assessment, *NA* not available, *NMSS* National Multiple Sclerosis Society, *PHQ-9* Patient Health Questionnaire, *PDQ-39* Parkinson’s Disease Questionnaire, *PI* principal investigator, *SR* sustained release, *SMA* Supplementary Motor Area

^a^ The recruitment status as found in the registration protocol of the trials

^b^ The actual enrollment (not the estimated enrollment) as found in the registration protocol of the trials

### Risk of bias in included studies

**Fig. 2:**
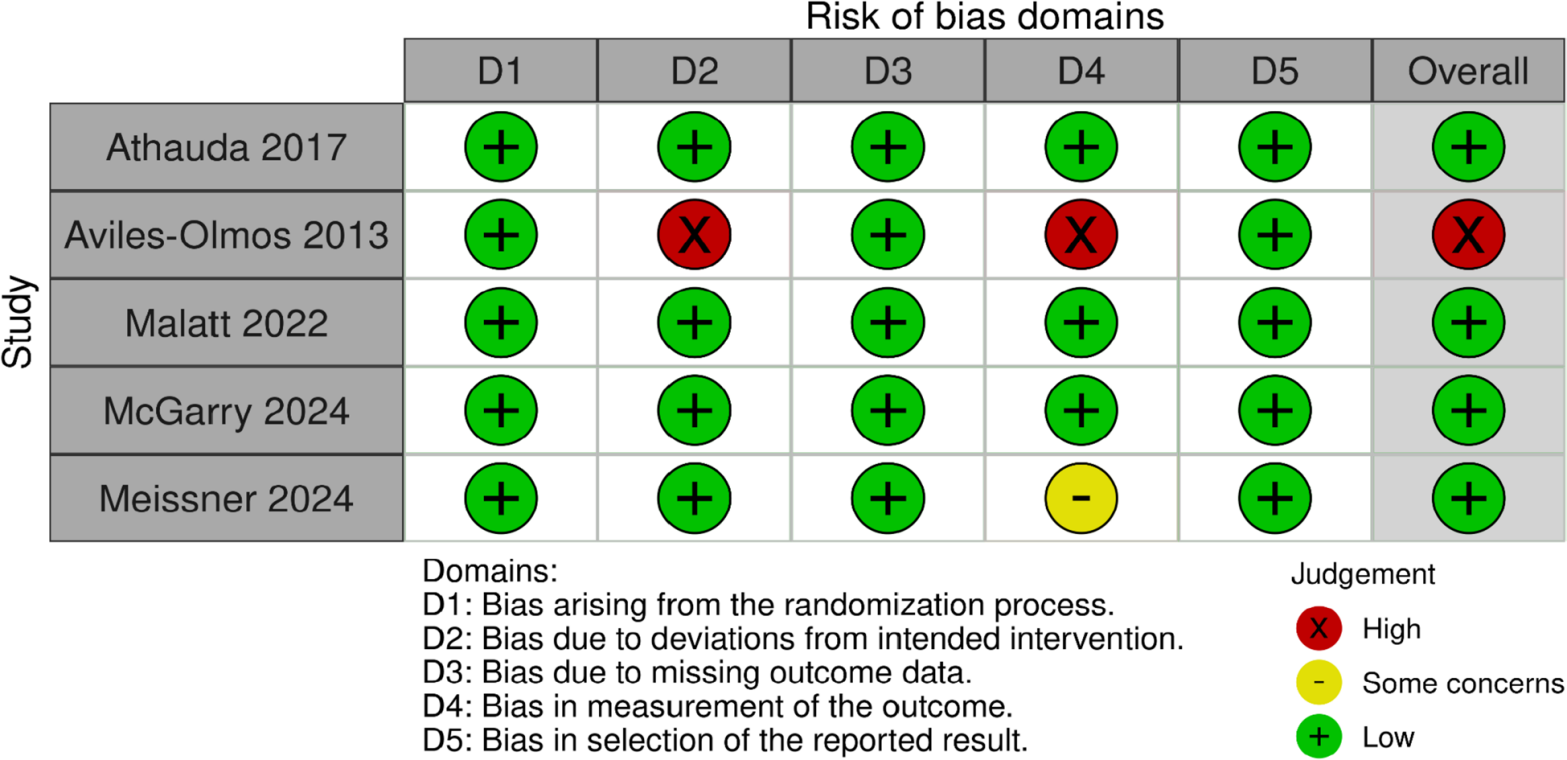
Risk of bias summary: The Authors' judgments about each risk of bias item in each included study

We used the RoB 2 tool to assess the risk of bias for each of the included studies. A summary of the risk of bias assessment is shown in Fig. 2. No significant risks of bias were observed regarding selection bias, including random sequence generation and missing data. A majority of the studies (4 out of 5) were considered to have a low overall risk of bias, while one report was assessed as having a high overall risk of bias. Except for Aviles-Olmos [28], which was single-blind, all studies were double-blind in terms of both evaluation and patient treatment. Aviles-Olmos [28] demonstrated a high risk of bias in two domains, including deviations from the intended intervention, and key clinical outcomes in PD patients were not initially measured for the study participants. In addition, Meissner 2024 has a limited management of key clinical outcomes [31].

### Primary outcomes results

#### MDS-UPDRS subscale Part III ON medication in endpoints

**Fig. 3:**
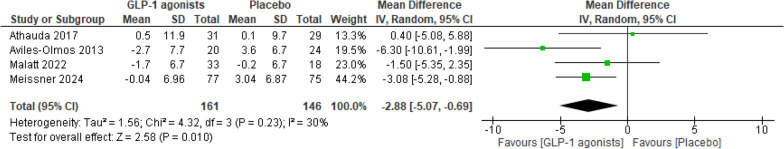
Analysis 1.1: GLP-1 Receptor Agonists vs. placebo in the outcome MDS-UPDRS Part III (on medication) in endpoints

Analysis of endpoint data on MDS-UPDRS Part III in the on-medication state showed a significant improvement in patients treated with GLP-1 receptor agonists compared to those given a placebo (MD = − 2.88; 95% CI − 5.07 to − 0.69; p = 0.01), with insignificant heterogeneity detected (I^2^ = 30%, p = 0.23) (Fig. 3).

Data on the on-medication state for MDS-UPDRS Part III were available from four studies. However, the study by McGarry et al. did not specify whether the MDS-UPDRS Part III assessments were conducted in the on-medication or off-medication state, and the authors did not respond to our inquiries for clarification [30].

**Fig. 4:**
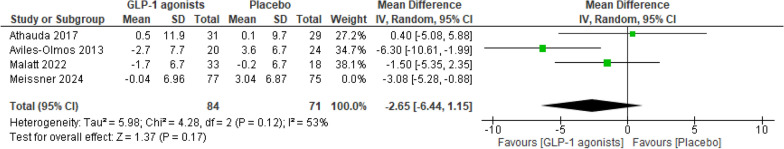
Analysis 1.1.1: GLP-1 receptor agonists vs. placebo, sensitivity analysis by excluding Meissner 2024

To evaluate the robustness of the evidence, we conducted a sensitivity analysis by excluding one study at a time from the meta-analysis to observe changes in the overall pooled results. A significant difference was detected only by excluding *Meissner 2024* [31]. After removing it from the meta-analysis, the effect size for the MDS-UPDRS Part III (on medication) in endpoints shifted to non-significance, indicating that this study may have had a disproportionate influence on the overall findings (MD = − 2.65; 95% CI − 6.44 to 1.15; p = 0.17) (Fig. 4).

#### MDS-UPDRS subscale Part III ON medication in midpoints

**Fig. 5:**
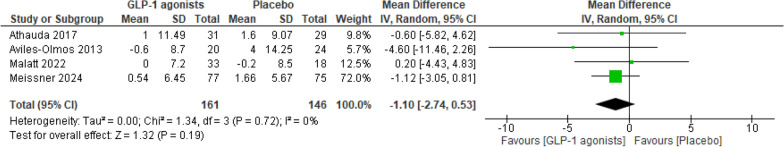
Analysis 1.2: GLP-1 receptor agonists vs. placebo in the outcome MDS-UPDRS Part III (on medication) in midpoints

An insignificant improvement in the MDS-UPDRS Part III in the on-medication state was detected when pooling effects of GLP-1 receptor agonists vs. placebo in midpoints assessment (MD = − 1.10; 95% CI − 2.74 to 0.53; p = 0.19) with no heterogeneity detected among studies (I^2^ = 0%, p = 0.72) (Fig. 5).

#### MDS-UPDRS subscale Part III OFF medication in endpoints

**Fig. 6:**
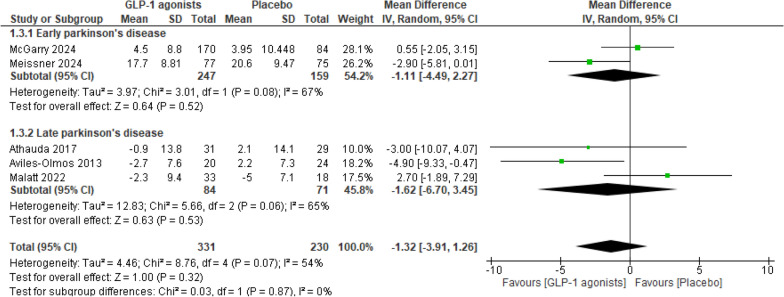
Analysis 1.3: GLP-1 receptor agonists vs placebo, in the outcome MDS-UPDRS Part III (off medication) in endpoints

Other insignificant improvements were also seen in the MDS-UPDRS Part III in the off-medication state total and subtotal analyses (Fig. 6). With enough trials for subgrouping in this outcome domain, we conducted a subgroup analysis based on the stage of PD as stated in the trials. Meissner 2024 limited the inclusion for subjects with onset of PD less than 3 years, while McGarry 2024 excluded PD subjects with more than 5 years of diagnosis [30, 31]. Therefore, these two studies are included in the “Early PD” subgroup. On the other hand, Aviles-Olmos 2013 only included patients who had PD for more than 5 years [28]. Moreover, the remaining two studies [27, 29] did not specify a duration of the disease for inclusion, and the time since diagnosis in these studies was much higher than in McGarry [30] and Meissner [31], as shown in Table 3 [27, 29].

Neither the total (sample size: 331 in the GLP-1 receptor agonists group and 230 in the placebo group) nor the subtotal (sample size: 247 and 159 in early PD and 84 and 71 in late PD, in the GLP-1 receptor agonists group and placebo group, respectively) improvements related to GLP-1 receptor agonists were significant (MD = − 1.32; 95% CI − 3.91 to 1.26; p = 0.32 for total comparison) (MD = − 1.11; 95% CI − 4.49 to 2.27; p = 0.52 for early PD subgroup) (MD = − 1.62; 95% CI − 6.70 to 3.45; p = 0.53 for late PD subgroup).

#### MDS-UPDRS subscale Part III OFF medication in midpoints

**Fig. 7:**
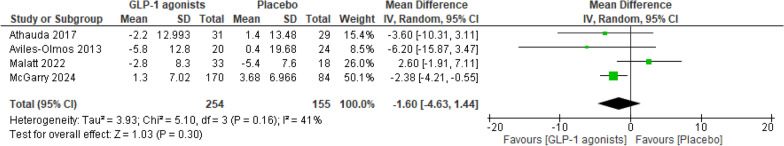
Analysis 1.4: GLP-1 receptor agonists vs. placebo in the outcome MDS-UPDRS Part III (off medication) in midpoints

The MDS-UPDRS Part III scores in the off-medication state in midpoints were not reported in Meissner 2024, and we contacted the authors with no response. Our analysis (Fig. 7) did not show a significant difference between the groups (MD = − 1.60; 95% CI − 4.63 to 1.44; p = 0.30), and no significant heterogeneity was observed (I^2^ = 41%, p = 0.16).

#### MDS-UPDRS Part IV in endpoints

**Fig. 8:**
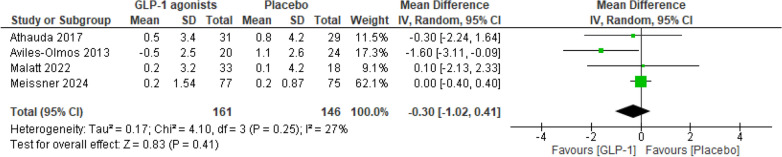
Analysis 1.5: GLP-1 receptor agonists vs. placebo in the outcome MDS-UPDRS Part IV in endpoints

The meta-analysis revealed no significant difference in MDS-UPDRS Part IV for individuals treated with GLP-1 receptor agonists compared to those receiving placebo (MD = − 0.30; 95% CI − 1.02 to 0.41; p = 0.41) (Fig. 8).

#### MDS-UPDRS Part II in endpoints

**Fig. 9:**
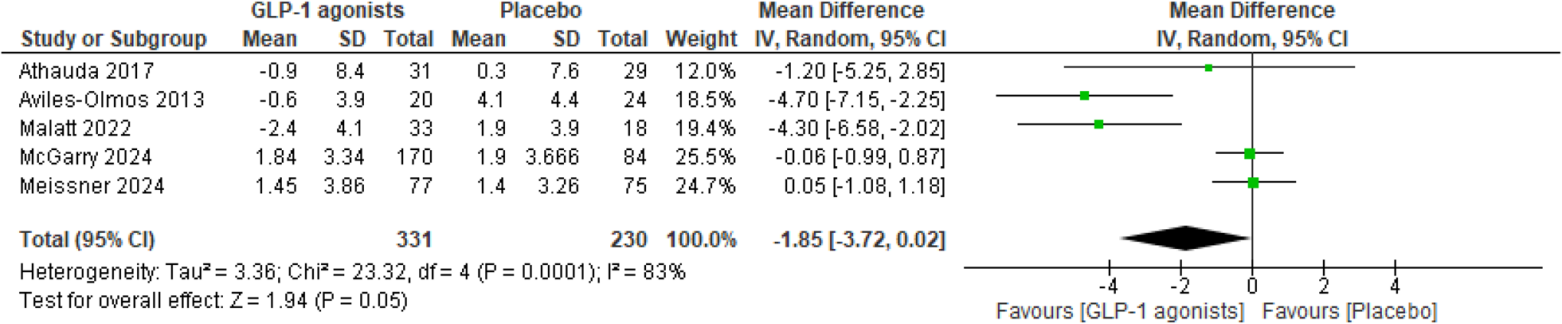
Analysis 1.6: GLP-1 receptor agonists vs. placebo, in the outcome MDS-UPDRS Part II in endpoints

As demonstrated in Fig. 9, an insignificant difference (MD = − 1.85; 95% CI − 3.72 to 0.02; p = 0.05) between the treatment and placebo groups was observed in MDS-UPDRS Part II, with high inter-study heterogeneity (I^2^ = 83%, p = 0.0001).

#### Dyspepsia

**Fig. 10:**
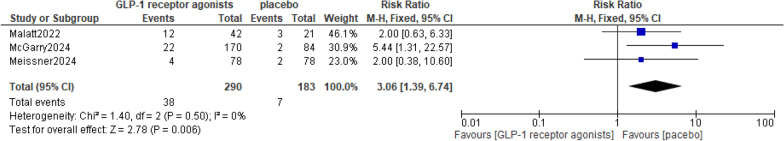
Analysis 1.7: GLP-1 receptor agonists vs. placebo, in the safety outcome: dyspepsia

Dyspepsia was reported in three studies [29–31], with a total sample of 473 participants (290 receiving GLP-1 receptor agonists and 183 receiving a placebo). The pooled estimate favored the placebo significantly (RR = 3.06; 95% CI 1.39 to 6.74; p = 0.006). There was no heterogeneity (I^2^ = 0%, p = 0.50), and a fixed-effect model was applied (Fig. 10).

#### Vomiting

**Fig. 11:**
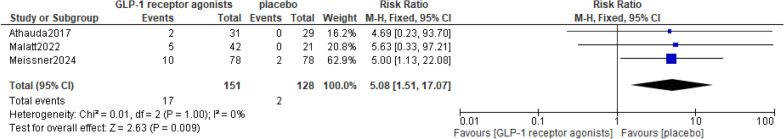
Analysis 1.8: GLP-1 receptor agonists vs. placebo, in the safety outcome: vomiting

Data on vomiting were also available from three studies [27, 29, 31], representing 323 participants (151 receiving GLP-1 receptor agonists and 128 receiving a placebo). According to our analysis (Fig. 11), significant differences favoring the placebo were found (RR = 5.08; 95% CI 1.51 to 17.07; p = 0.009).

#### Diarrhea

**Fig. 12:**
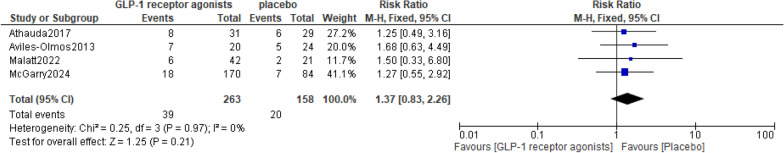
Analysis 1.9: GLP-1 receptor agonists vs. placebo, in the safety outcome: diarrhea

In consistency with the previous safety outcomes, diarrhea was more frequent in the GLP-1 receptor agonists group (Fig. 12).

#### Constipation

**Fig. 13:**
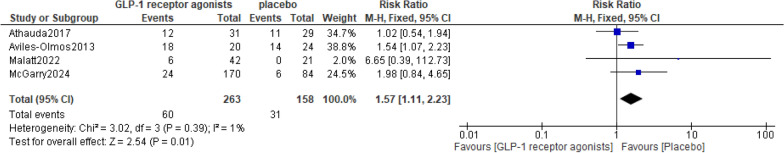
Analysis 1.10: GLP-1 receptor agonists vs. placebo, in the safety outcome: constipation

The pooled effect estimate shows a significant difference between the groups, favoring the placebo with an RR of 1.57 and a p-value of 0.01 (Fig. 13).

#### Headache

**Fig. 14:**
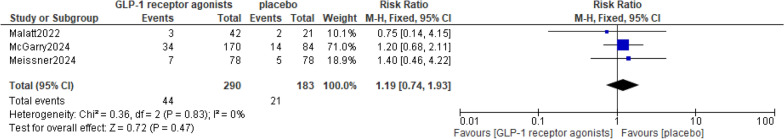
Analysis 1.11: GLP-1 receptor agonists vs. placebo in the safety outcome: headache

Similarly to diarrhea, headache has an insignificantly higher incidence in the intervention group (RR = 1.19; 95% CI 0.74 to 1.93; p = 0.47) (Fig. 14).

#### Fatigue

**Fig. 15:**
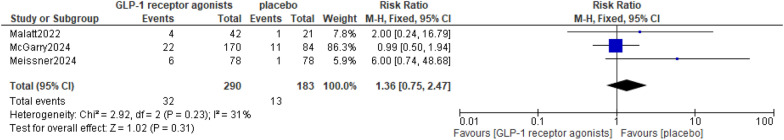
Analysis 1.12: GLP-1 receptor agonists vs. placebo in the safety outcome: fatigue

The pooled analysis indicated no significant difference between the treatment and placebo groups in fatigue; however, it favored the placebo (Fig. 15).

### Secondary outcomes

#### NMSS

**Fig. 16:**
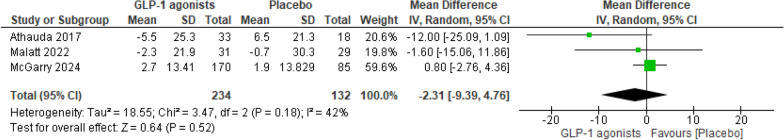
Analysis 2.1: GLP-1 receptor agonists vs. placebo, NMSS

The analysis did not demonstrate a statistically significant difference in the NMSS for patients receiving the treatment compared to those receiving a placebo (MD = − 2.31; 95% CI − 9.39 to 4.76; p = 0.52). There was no heterogeneity (I^2^ = 42%, p = 0.18), and a random-effects model was employed (Fig. 16).

#### PDQ‐39

**Fig. 17:**
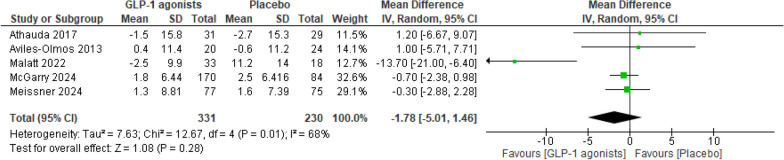
Analysis 2.2: GLP-1 receptor agonists vs. placebo, PDQ-39

The meta-analysis (Fig. 17) revealed no significant change in the PDQ-39 between the treatment group and the placebo group (MD = − 1.78; 95% CI − 5.01 to 1.46; p = 0.28).

#### LED

**Fig. 18:**
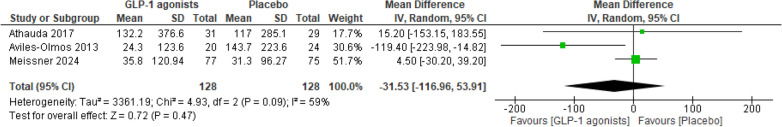
Analysis 2.3: GLP-1 receptor agonists vs. placebo, LED

Similarly to the other non-motor symptoms, the pooled analysis showed an insignificant improvement in the LED in the treatment groups compared to the placebo (MD = − 31.53; 95% CI − 116.96 to 53.91; p = 0.47) (Fig. 18).

## Discussion

### Summary of main results

This meta-analysis evaluated the effects of GLP-1 receptor agonists on key clinical outcomes, specifically the MDS-UPDRS subscales (Parts III, IV, and II), along with the frequency of adverse events. We found statistically significant improvements in the MDS-UPDRS Part III ON medication subscale in subjects receiving GLP-1 receptor agonists compared to the placebo group in endpoints. Notably, the results from the trial by Meissner et al. were the only ones that, when excluded, altered the significance of the results [31]. However, no significant improvements were observed in the MDS-UPDRS Parts II and IV. Regarding safety, the GLP-1 receptor agonists group reported a higher frequency of all adverse events, significantly in dyspepsia, vomiting, and constipation, with non-significant differences in diarrhea, headache, and fatigue.

### Context of our findings

Therapies for PD have been devised based on the principle that PD is underlined by a deficiency in dopamine within the striatum. Treatments included dopamine agonists that stimulate neuronal dopamine receptors, such as levodopa; monoamine oxidase-B (MAO-B) inhibitors that inhibit metabolism of dopamine in the brain, such as selegiline and rasagiline; and catechol-*O*-methyl transferase (COMT) inhibitors that inhibit degradation of levodopa in the periphery, such as entacapone and opicapone [50].

Problems in PD therapies include the short half-life and fluctuant plasma levels of levodopa, the precursor of dopamine [51]; variable effects of MAO-B [50]; and the short duration of entacapone and opicapone [52], as well as other troubles that necessitated the search for a viable different option.

The rationale behind the use of GLP-1 receptor agonists in the management of PD is supported by the fact that T2DM patients experience a more severe form of PD than non-diabetic patients. They show significantly more severe motor symptoms, greater total non-motor symptoms, poorer cognitive scores, and a greater need for greater amounts of dopaminergic medication. Moreover, over time, patients with T2DM have significantly faster progression of motor symptoms, worse depression scores, and are more likely to develop substantial gait impairment and mild cognitive impairment than patients without T2DM [53, 54].

GLP-1 receptor agonists’ potential therapeutic effects appear to target key pathological mechanisms of PD directly. Preclinical studies have shown that GLP-1 receptor activation reduces neuroinflammation by suppressing pro-inflammatory cytokines such as TNF-α and inhibiting glial cell activation [55]. Additionally, these agents mitigate oxidative stress through upregulation of antioxidant pathways, including NRF2 (nuclear factor erythroid 2-related factor 2), enhance mitochondrial function, and reduce pathological α-synuclein aggregation [56, 57]. These multifaceted mechanisms suggest that GLP-1 receptor agonists may offer disease-modifying benefits in PD, beyond their established metabolic effects.

While previous studies have reported a reduced risk of developing PD in *diabetic patients* using GLP-1 receptor agonists, likely due to improved glucose metabolism and reduced neuroinflammatory burden, it is important to note that the trials assessing their efficacy in managing PD and included in our analysis applied a diagnosis of diabetes as an exclusion criterion. This creates a gap in the evidence. Future investigations should include diabetic populations with PD to properly assess the efficacy profile of GLP-1 receptor agonists and determine whether the observed improvement in the MDS-UPDRS Part III score (MD = − 2.88; p = 0.01) from our analysis is reproducible in such populations.

Beyond the CNS, and in another diabetes-related disorder, GLP-1 receptor agonists have shown great promise in diabetic peripheral neuropathy (DPN), a common complication of diabetes marked by nerve damage caused by chronic hyperglycemia and oxidative stress. The metabolic homeostasis that GLP-1 receptor agonists can induce has even been suggested to “reverse” nerve morphological abnormalities in DPN [58]. The mechanisms proposed to explain their benefits in peripheral nerves closely parallel those implicated in their neuroprotective effects in the brain [59]. These parallels further support the need to investigate the therapeutic potential of GLP-1 receptor agonists in diabetic patients with PD, who may benefit from both central and peripheral neuroprotection.

### Agreements and disagreements with other studies or reviews

A similar Cochrane review was conducted in 2020, at a time when only two trials had been published investigating the role of GLP-1 receptor agonists in PD [27, 28, 60]. However, that review did not pool the findings from these studies in a meta-analysis, which limits direct comparison with our results. Since then, additional trials have been published, further contributing to the growing body of evidence on the potential role of GLP-1 receptor agonists in PD management [29–31].

A more recent meta-analysis included the same number of trials as ours and reported similar findings with regard to motor symptoms, assessed by MDS-UPDRS Part III. However, our study provides a significantly more comprehensive evaluation. Notably, unlike Albuquerque and colleagues, our review included both published and ongoing studies, offering broader insights into the current landscape and future directions of GLP-1 receptor agonist research in PD. Additionally, our meta-analysis pooled a wider range of outcomes, encompassing not only motor symptoms but also safety outcomes, an aspect not addressed in their analysis. We evaluated six distinct gastrointestinal and systemic adverse events, which were designated as primary outcomes in our review. Furthermore, in our approach, we analyzed data at multiple time points (midpoints and endpoints), allowing a longitudinal assessment of efficacy that was not explored in previous reviews [61].

An earlier general systematic review on the neuroprotective role of GLP-1, which included both clinical and preclinical trials in PD, Alzheimer’s disease (AD), mood disorders, and other conditions, concluded—based on preclinical data—that GLP-1 has a substantial role in neuroprotection [62]. This was thought to occur through mechanisms such as the restoration of neurite outgrowth, increased neurotrophic factors, and strengthening of the blood–brain barrier. However, the clinical evidence included in that review was insufficient to draw conclusive interpretations about GLP-1’s potential in individuals with neurodegenerative diseases.

In AD, some studies have suggested that the GLP-1 receptor agonist liraglutide may slow disease progression [63]. However, the mechanisms behind these effects remain poorly understood. A recent systematic review assessing GLP-1 receptor agonists in AD found that, while these agents do not appear to significantly improve cognitive function via effects on amyloid-β and tau biomarkers, they may still offer metabolic and neuroprotective benefits [64].

In the context of amyotrophic lateral sclerosis (ALS), another neurodegenerative disorder, two recent cross-sectional case–control studies examined the role of metabolic biomarkers, including GLP-1, in the progression and pathogenesis of ALS [65, 66]. These studies notably found elevated levels of GLP-1 in ALS patients compared to healthy controls. However, GLP-1 showed insignificant correlations with all subscales of the ALS functional scale (ALSFRS-R), although a weak correlation was observed with the total ALSFRS-R score (r = 0.205; p = 0.032) [66]. This suggests that while GLP-1 levels are altered in ALS, their clinical relevance to functional status remains uncertain.

These mixed findings of GLP-1 and its receptor agonists research in neurodegenerative diseases underscore the complexity of their role, highlighting the need for further research to clarify their potential impact on the pathology and prognosis of such conditions.

### Limitations

Several limitations must be considered when interpreting the findings of this review. First, the sample sizes in the included trials were relatively small, which may have limited the power to detect significant differences in outcomes. Although all of the trials were randomized, some with small cohorts failed to fully balance baseline characteristics between experimental and control groups despite the randomization [27, 28]. Differences in baseline characteristics of trial arms may affect intervention response and disease progression [28], potentially impacting or skewing the results of the trials.

Additionally, the small sample sizes in the included studies led to some questionable observations. For instance, in two included trials, younger PD subjects in the placebo group exhibited greater and more rapid motor deterioration, as measured by the MDS-UPDRS Part III subscale, compared to their older counterparts [30, 38]. This contrasts with most PD literature, which suggests that younger patients generally have better compensatory resiliency and slower disease progression [67–69]. However, due to the limited number of participants, plausible explanations for this observation remain unclear.

Moreover, the self-administration of GLP-1 receptor agonists via subcutaneous injection in all of the included trials adds an element of uncertainty regarding the validity of the results. Noncompliance is a common issue with self-administered treatments. Additionally, the use of subcutaneous injections of GLP-1 receptor agonists via a pen may represent an unfamiliar and potentially challenging route of administration for some participants.

Notably, none of the included trials investigated newer GLP-1 compounds such as tirzepatide, a dual GIP/GLP-1 receptor agonist, or semaglutide. These agents have demonstrated particular promise, with semaglutide showing a 40% reduction in AD risk in subjects with T2DM compared to other GLP-1 receptor agonists in a previous cohort study [70]. This profile opens promising avenues for investigation as management strategies in neurodegenerative diseases, including PD.

Another factor to consider when interpreting our results with caution is the inconsistency in the endpoints across the included studies, with follow-up periods ranging from 44 to 60 weeks [27–31]. This variation in study duration may introduce inconsistency across the studies. In addition, this variability could introduce biases related to our selection of specific time points for data pooling, which affects the comparability of the studies [60].

### Strengths

Despite these limitations, this review benefits from several strengths. We conducted a thorough search of both published and gray literature to ensure the inclusion of all relevant clinical trials on this topic. Additionally, to minimize errors and bias, nearly every step of the review process was performed by at least two reviewers. Furthermore, rigorous methodological standards, including adherence to the Cochrane Handbook for Systematic Reviews, were followed to ensure robustness in our approach. For instance, we carefully addressed issues related to the unit of analysis, such as the multiple treatment arms in *McGarry 2024*, to avoid errors in the data synthesis process (see “Unit of analysis issues” section Unit of analysis issues) [30, 33]. Unit of analysis problems are common in meta-analyses [71]. Moreover, four of the five included studies were of high quality, with a low risk of bias across most domains, which enhances the confidence in the validity of our findings.

## Conclusions

Our analyses yielded inconclusive results regarding the effect of GLP-1 receptor agonists in PD treatment, primarily due to the limited number of trials and participants. Given the questionable observations in the studies and the unclear mechanisms of action, it is currently not recommended to use or attempt GLP-1 receptor agonists in clinical settings for PD outside of research contexts. Further double-blinded, placebo-controlled multicenter trials are needed to provide more definitive clinical insights. Larger sample sizes are crucial to account for baseline imbalances between study groups and to better elucidate the specific impact of GLP-1 receptor agonists on both the symptoms and progression of PD. Furthermore, the longest follow-up period in the included studies of our review was 60 weeks, which may not be sufficient for evaluating long-term effects in a disease as chronically progressive as PD. It is worth noting that one of the ongoing studies is planned to extend over a 4-year period, which may provide more comprehensive data on the long-term effects of GLP-1 receptor agonists. These future studies should address the uncertainties raised in previous evidence, particularly regarding the role of GLP-1 in disease progression, its mechanism of action, and its safety profile, to further clarify the potential of GLP-1 receptor agonists as a therapeutic option in PD.

## Data Availability

Data is provided within the manuscript.
